# Primary Renal Lymphoma: Report of 32 Cases—A Retrospective Multicenter PLRG Analysis

**DOI:** 10.3390/biomedicines13030548

**Published:** 2025-02-21

**Authors:** Magdalena Witkowska, Joanna Romejko-Jarosińska, Agnieszka Giza, Joanna Drozd-Sokołowska, Damian Mikulski, Janusz Hałka, Anna Morawska-Krekora, Ewa Paszkiewicz-Kozik, Kamil Wdowiak, Dariusz Wołowiec, on behalf of the Polish Lymphoma Research Group

**Affiliations:** 1 Department of Hematology, Medical University of Lodz, 93-510 Lodz, Poland; 2 Department of Lymphoid Malignancies, Maria Sklodowska-Curie National Research Institute of Oncology, 02-781 Warsaw, Poland; 3 Department of Haematology, Jagiellonian University, 31-007 Krakow, Poland; 4 Department of Hematology, Transplantology and Internal Diseases, Medical University of Warsaw, 02-097 Warsaw, Poland; 5 Department of Biostatistics and Translational Medicine, Medical University of Lodz, 93-510 Lodz, Poland; 6 Department of Hematooncology, Copernicus Memorial Hospital, 93-513 Lodz, Poland; 7 Department of Hematology, Internal Medicine and BMT, Clinical Hospital of the Ministry of Internal Affairs and Administration with the Warmia-Mazury Oncology Center, 10-228 Olsztyn, Poland; 8 Department of Oncology, University of Warmia and Mazury, 10-719 Olsztyn, Poland; 9 Department of Hematology and Internal Diseases, Ludwik Rydygier’s Hospital, 31-820 Krakow, Poland; 10 Department of Internal Diseases and Oncological Chemotherapy, Faculty of Medical Sciences, Medical University of Silesia, 40-055 Katowice, Poland; 11 Department and Clinics of Hematology Cellular Therapies and Internal Medicine, Wrocław Medical University, 50-367 Wrocław, Poland

**Keywords:** extranodal lymphomas, lymphoma, primary renal lymphoma, rare lymphomas, renal lymphoma

## Abstract

**Introduction**: Primary renal lymphoma is extremely rare, accounting for less than 1% of all lymphomas. **Objectives**: The aim of this study was to describe a group of patients with primary renal lymphoma diagnosed in hematology and oncology centers aligned with the Polish Lymphoma Research Group (PLRG). **Patients and methods**: This was a retrospective analysis of adult patients with primary renal lymphoma diagnosed at PLRG centers. **Results**: Thirty-two patients were diagnosed in seven centers over 24 years (2000–2023). The most common type of lymphoma was diffuse large B-cell lymphoma (DLBCL). The median progression-free survival (PFS) after first-line treatment was 2.1 (95% CI: 1.07–4.18) years, while the median overall survival (OS) was 6.3 (95% CI: 1.82–6.34) years. The median age at diagnosis was 63.3 years old, and 59.4% of the patients were females. In multivariate Cox regression for PFS, only creatinine > 1.5 mg/dL (HR 10.2, 95% CI: 2.08–50.09, *p* = 0.004) and hemoglobin (Hgb) < 10 g/dL (HR 8.39, 95% CI: 1.88–37.49) were associated with inferior PFS. Patients who achieved complete remission (CR) after first-line of treatment had significantly longer PFS (median 4.18, 95% CI: 2.02–4.18 vs. median 0.73, 95% CI: 0.50–0.79, *p* = 0.004) and OS (median not reached vs. median 1.49, 95% CI: 0.43–6.33, *p* = 0.001). Patients treated with nephrectomy had longer OS (median not reached vs. median 5.07, 95% CI: 1.32–5.08, *p* = 0.05). However, in multivariate Cox regression for OS, only hypoalbuminemia was an independent factor for inferior survival (HR 5.44, 95% CI: 1.12–26.38, *p* = 0.04). **Conclusions**: Primary renal lymphoma is an extremely rare type of lymphoma with a poor prognosis. The prognosis may improve with future advances in treatment, including nephrectomy.

## 1. Introduction

Malignant lymphomas can arise in extranodal organs, initially without infiltration of the lymphoid organs, like lymph nodes, spleen, Waldeyer’s ring or thymus. These lymphomas are called primary extranodal lymphomas, and they account for about one-third of all non-Hodgkin lymphomas (NHL) [1]. Secondary renal involvement in NHL is relatively common in advanced, disseminated stages and can be seen in up to 30–60% of NHL patients [2]. Primary renal lymphoma (PRL) is, however, very rare. The incidence of PRL remains elusive, since a significant proportion of cases probably remain undiagnosed due to asymptomatic course or unspecific symptoms.

PRL is defined as the primary, isolated involvement of one kidney or both kidneys without affecting/involvement of other organs, and with the absence of extensive nodal disease [3]. However, involvement of PRL disease may be observed. A definitive diagnosis may be made solely by histological examination of the renal biopsy specimen. PRL is usually diagnosed in middle-aged or elderly patients. Most cases are high-grade lymphomas, and the great majority of the cases described so far are of B-cell origin, with only few T-cell lymphomas reported [4]. Patients may be asymptomatic or can present with back pain, weight loss, hematuria and palpable abdominal mass. It has been suggested that flank pain is one of the most common symptoms of PRL [5].

In the reports published so far, the most common therapy for patients with PRL was chemotherapy, most frequently six to eight cycles of the CHOP regimen (cyclophosphamide, daunorubicin, vincristine and prednisone) in combination with rituximab—an anti-CD20 monoclonal antibody [6]. Reported 1-year mortality rates for PRL were as high as 75%, with median survival time ranging between 8 months and 3 years [7]. The 5-year overall survival (OS) rate did not exceed 50% [8].

To date, more than 700 cases have been reported in the literature. Due to the limited number of studies, which report few PRL patients, the data on PRL are scarce. The aim of our study was to analyze the medical history of PRL patients diagnosed and treated at Polish Lymphoma Research Group (PLRG) centers in the years 2000–2023, examining incidence, sex distribution, histological diagnosis, risk factors, different therapies and their impact on progression-free survival (PFS) and OS.

## 2. Patients and Methods

We retrospectively analyzed 32 patients with PRL, hospitalized in seven Polish hematological/oncological centers between January 2000 and December 2023. Data were collected from the local medical records.

Cases diagnosed between 2000 and 2007 were classified according to the World Health Organization (WHO) classification from 2001 [9], those diagnosed between 2008 and 2022 were classified according to the WHO 4th edition classification [10], and those diagnosed in 2023 were classified according to the WHO 5th edition classification [11].

PRL was defined as involving one kidney or two kidneys, with the absence of known extrarenal organ involvement and the absence of extensive lymph node involvement (sizes of more than 4 cm were excluded from the study). However, single involvement of PRL disease may be present [3].

According to current Lugano criteria, complete response (CR) was defined as a complete resolution of all target lesions by computer tomography (CT) scans, and, when applicable, with complete normalization of fluorodeoxyglucose (FDG)-positron emission tomography (PET) uptake in all areas (Deauville score of 1–3). Partial response (PR) was defined as a decrease in the sum of the products of diameters (SPD) of target lesions by ≥50%, with no increase in the size of any lesion and no appearance of new lesions [12].

The study was performed in accordance with the local ethics committee. The collected data did not include any personally identifiable information; therefore, informed consent from the patients was not required.

## 3. Statistical Analysis

The nominal variables were presented as frequencies and percentages (of all patients with data available), whereas continuous variables were reported as mean ± standard deviation or median with interquartile range depending on the analyzed variable distribution. The median follow-up was calculated using the reverse Kaplan–Meier estimator. The median PFS and OS were calculated using the Kaplan–Meier method. The median follow-up was calculated using the reverse Kaplan–Meier estimator. Prognostic factors were initially evaluated using univariate and multivariate Cox Proportional Hazards models. Kaplan–Meier curves were visualized and compared using the log-rank test. *p*-values lower than 0.05 were considered statistically significant. All statistical analyses were conducted using Statistica version 13.1 (TIBCO, Palo Alto, CA, USA).

## 4. Results

Thirty-two patients were included in the study. The median age at the diagnosis was 63.3 (53.8–69.9). Most patients (59.4%) were females. At diagnosis, the most frequent complaint was abdominal or lumbar pain (28.1%), followed by systemic symptoms (21.9%). A smaller proportion reported hematuria (15.6%), two patients (6.3%) reported fatigue as the main complaint, one (3.1%) presented with symptoms of hyponatremia, and one (3.1%) with local lymphadenopathy. However, it is noteworthy that 18.8% of lymphoma cases were detected incidentally in ultrasonography (USG) or CT scans performed for other reasons. Overall, systemic symptoms were present in half of the patients (50.0%), and local lymph node involvement was found in more than half of the cases (59.4%). Fifteen patients (46.9%) had lymphoma infiltration of tissue surrounding the kidneys. Notably, the left kidney emerged as the most common site of primary tumor localization (50.0%), with 25% in the right kidney, and 6.3% in both kidneys (see Table 1). The mean tumor’s largest diameter was 9.5 ± 4.4 cm. Eighteen (56.3%) patients had the largest tumor diameter > 5 cm. Elevated serum lactate dehydrogenase (LDH) activity was observed in 18 (56.3%) patients, and elevated serum b2-microglobulin (β-2M) concentration was observed in seven (21.9%) patients. Five (15.6%) patients had moderate to severe anemia (hemoglobin Hgb < 10 g/dL), seven (21.9%) had creatinine levels > 1.5 mg/dL and 10 (31.3%) patients developed hypoalbuminemia.

The diagnosis was confirmed by histopathological exam: 11 patients had nephrectomy, 10 patients had surgical kidney biopsy and 11 had regional lymph node biopsy.

The histopathological diagnoses were as follows: diffuse large B-cell lymphoma (DLBCL) in 18 patients (56.3%), followed by marginal zone lymphoma (MZL) in eight patients (25%), four patients (12.5%) with follicular lymphoma (FL), one patient with Hodgkin lymphoma (HL) and one with high-grade B-cell lymphoma (HGBL). The detailed characteristics of the study cohort are provided in Table 1.

Treatment strategies varied, with 65.6% of patients having received immunochemotherapy (21 patients) as the sole intervention and 25.0% undergoing a combination of immunochemotherapy and radical surgery as the primary therapeutic approach (eight patients). Two patients with DLBCL underwent only radical surgery, and one patient had a partial renal resection in combination with immunochemotherapy. During chemotherapy, a granulocyte colony-stimulating factor (G-CSF) was administered to 15 (46.9%) patients. After the first-line treatment, all 32 patients were assessed for response: 21 (65.6%) patients achieved CR, five (15.6%) PR and four (12.5%) experienced progressive disease (PD).

**Figure 1:**
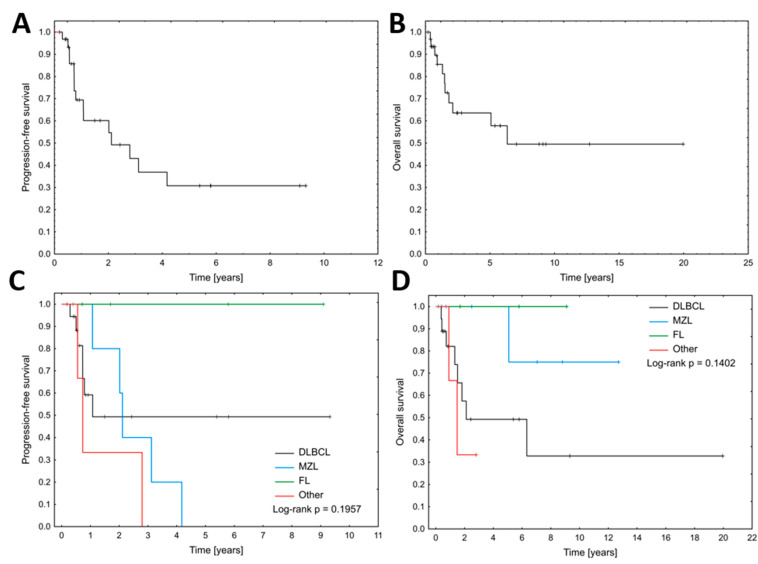
Median progression-free survival (**A**) and overall survival (**B**) for all 32 cases of primary renal lymphoma. Median progression-free survival (**C**) and overall survival (**D**) between histopathological lymphoma types.

**Figure 2:**
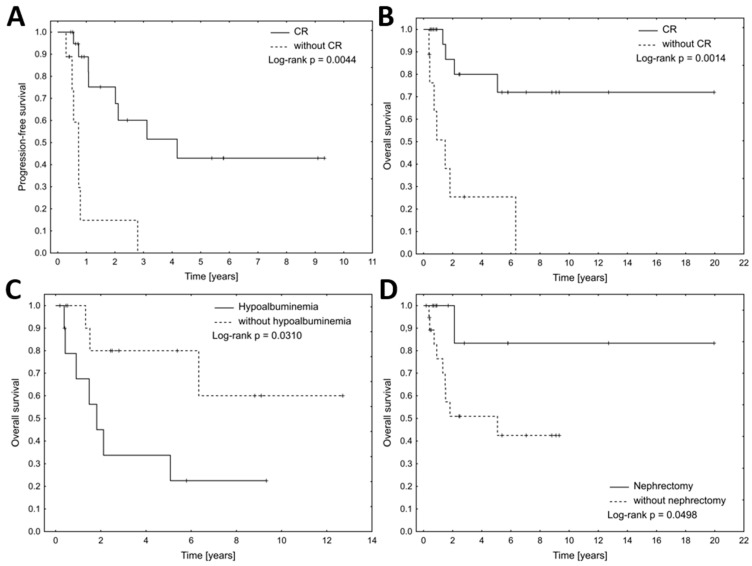
Median progression-free survival (**A**) and overall survival (**B**) for patients with and without complete remission. Median overall survival (**C**) for patients with and without hypoalbuminemia. Median overall survival (**D**) for patients with and without nephrectomy.

With a median follow-up of 5.39 years (95% CI: 2.43–8.81), 17 out of 32 patients experienced relapse or progression between 3 to 32 months after the end of the first-line treatment. The median PFS in the whole study cohort was 2.11 years (95% CI: 1.07–4.18). Ten patients died (nine from the lymphoma, one from causes unrelated to the lymphoma). Eighteen patients remain alive, with data missing for four patients. Median OS was 6.33 (95% CI: 1.82–6.34) years (Figure 1A,B). There were no statistically significant differences in median PFS and OS between histopathological lymphoma types (*p* = 0.2 and *p* = 0.14, respectively) (Figure 1C,D). In multivariate Cox regression for PFS, only serum creatinine concentration > 1.5 mg/dL (HR 10.2, 95% CI: 2.08–50.09, *p* = 0.004) and Hgb < 10 g/dL (HR 8.39, 95% CI: 1.88–37.49) were significantly associated with inferior PFS (Table 2). Additionally, patients who achieved CR after the first line of treatment had longer PFS (median 4.18, 95% CI: 2.02–4.18 vs. median 0.73, 95% CI: 0.50–0.79, *p* = 0.004) and OS (median not reached vs. median 1.49, 95% CI: 0.43–6.33, *p* = 0.001) (Figure 2A,B). Furthermore, patients who underwent nephrectomy, either as the only therapeutic procedure or in association with immunochemotherapy, had significantly longer OS than patients who received only conservative treatment (median not reached vs. median 5.07, 95% CI: 1.32–5.08, *p* = 0.0498, Figure 2D), however, in multivariate Cox regression for OS, only hypoalbuminemia was associated with inferior survival (HR 5.44, 95% CI: 1.12–26.38, *p* = 0.04, Figure 2C).

In our cohort, central nervous system (CNS) relapses occurred in five (27.8%) patients, all of which were lethal. Three patients had high CNS-IPI (international prognostic index) scores and received CNS prophylaxis, one had an intermediate CNS-IPI score and one had a low CNS-IPI score. Patients with low and intermediate scores did not receive any kind of CNS prophylaxis. CNS prophylaxis procedures were given only to patients with high CNS-IPI scores. Two patients had three cycles of intrathecal treatment of methotrexate alone (both had CNS relapse). Two patients had two cycles of high-dose intravenous methotrexate treatment given at the end of the immunochemotherapy (one had CNS relapse).

## 5. Discussion

Since the first report in 1989, where the first patient with PRL was diagnosed by open kidney biopsy, more than 700 cases have been reported in the medical literature [13]. During the literature search, Stallone et al. [14] reported 29 cases of PRL up to the year 2000 [15,16,17,18]. PRL is an extremely rare type of lymphoma, with an estimated incidence of 0.7% of all extranodal lymphomas [16]. The present study reports 32 cases of PRL diagnosed in seven PLRG centers in Poland between 2000 and 2023. The biggest studies so far reported 121 cases by Lee et al. [19] and 599 in a population-based analysis using the SEER program [20]. According to this analysis, PRL had male predominance, with a male:female (M:F) ratio of 1.6:1 (72:45; unknown, four patients), unlike in our study, where 59.4% of all cases were females. The median age of the patients in the group of 121 patients was 55 years, while in our cohort, the patients were slightly older, with a median age of 63.3 years.

According to published data, the most common histological subtype of PRL is DLBCL, with MZL as the second most common [21]. In our group, the histological subtypes were similarly distributed with a predominance of DLBCL (56.3% of cases), followed by MZL in 21.9%. Another common type of PRL was FL, identified in 12.5% of cases. Moreover, there was one case of HL and one of HGBL. This distribution aligns with previous reports, which described individual cases of other types of lymphomas [22,23,24].

In previously reported series, most patients presented with abdominal and flank pain (62%). Fever of unknown etiology was the most prevalent symptom in younger patients (56%), while weight loss and gross hematuria were the main symptoms in older patients (37%) [22]. In our group, the initial symptoms were similar but were reported less frequently. At diagnosis, 28% of our patients complained about abdominal or flank pain and 22% had B-symptoms, including unexplained fever, weight loss and night sweats. Hematuria was less frequently reported (15.6%). We cannot exclude the possibility that this discrepancy results from the limitations of the retrospective character of the study, with incomplete medical records concerning relevant symptoms reported by the patients. It was observed that the symptoms may be more severe. In a study by Lubas et al., renal infiltration of mantle cell lymphoma (MCL) caused subacute membranoproliferative glomerulonephritis and acute kidney injury [25].

As the kidney does not contain lymphoid tissue, the primary renal localization of lymphoma was initially debated. Although PRL is usually suspected after completing a series of imaging exams, it can be diagnosed definitively by kidney biopsy. While contrast-enhanced CT is the best method for diagnosing a kidney tumor, ultrasound is usually the first imaging technique used by physicians when suspecting abdominal pathology. In two-thirds of patients, CT scans show isodense or hyperdense bilateral masses with irregular borders or encapsulated masses, along with retroperitoneal lymphadenopathy [26]. Tumor diameters can vary, mostly ranging from 1 to 4.5 cm [24]. In our population, tumor sizes were much larger, with a mean size of 9.5 ± 4.4. Moreover, 18 patients (56.3%) had a tumor size greater than 5 cm. We also observed that local lymph node involvement was common (57.9%), typically affecting the retroperitoneal ones. Fifteen patients (46.9%) had lymphoma infiltration into the surrounding kidney tissue. Literature data suggest that local lymph node infiltration may be seen in up to 25–30% of PRL patients [16,17,26]. This data suggest that our group may have been diagnosed at a later stage of the disease. It was consistent with our findings that the asymptomatic onset of the disease was more frequent in our series compared to previously published ones.

According to previously published data, PRL is usually unilateral but may also be bilateral in a proportion of patients [24]. In our series, two cases out of 32 cases (6.25%) had bilateral involvement. In unilateral cases, left kidney involvement predominated (50.0%), with 25% involving the right kidney. Previous data showed that bilateral PRL is usually observed in younger patients, with these studies also showing shorter survival times in patients with bilateral PRL compared to those with unilateral PRL [24]. In our study, however, different data were observed: patients with bilateral involvement were older. Nevertheless, consistent with previous reports, survival in our patients with bilateral renal involvement was short due to the progressiveness of the disease.

Because of the rarity of PRL, no clinical studies have been specifically designed for its treatment, nor are there formal recommendations for this group of NHL. Therefore, the clinical practice is usually based on the results of trials designed for the general lymphoma population and the corresponding histological subtypes. That said, immunochemotherapy remains the cornerstone of treatment for B-cell PRL, with R-CHOP-like regimens being recommended by experts for PRL with aggressive histology [5]. The role of surgical treatment in PRL has not been well established, although Chen X. et al. reported better outcomes for patients who underwent surgery [24]. The utility of radiotherapy in PRL has not been firmly established either. Treatment strategies for our patients varied. Out of the 32 patients, 31 (96.9%) were treated with chemotherapy, either alone or in combination with immunotherapy. A total of 65.6% of patients received immunochemotherapy as the sole intervention, while 25.0% underwent a combination of immunochemotherapy and radical surgery as the primary therapeutic approach. All patients responded to this treatment. Two patients (6.3%) underwent nephrectomy alone, and another received a partial nephrectomy in association with immunochemotherapy. Our data showed that nephrectomy was associated with a significant improvement in OS. Further investigation should be conducted to determine whether histological sub-type and rituximab have any impact on this conclusion.

According to previously published data, patients with NHL with renal involvement are at a higher risk of CNS relapse. In a retrospective study by Villa et al. [27], among 2656 DLBCL patients, 52 had primary and secondary renal involvement at diagnosis, with 20 (36%) experiencing CNS relapse shortly after the end of treatment. Similar findings were observed in our study. CNS relapses were reported in five (27.8%) patients, all of which were lethal. Although our cohort was too small for statistical conclusions, our results strongly suggest that patients with PRL are at an elevated risk of CNS involvement. Additionally, based on our results, two out of five patients who relapsed did not have high CNS-IPI scores. We suspect that CNS-IPI is not sufficient for the decision of prophylaxis in this group of patients. In our opinion, due to poor prognosis, CNS should be additionally examined in the diagnosis of PRL by CNS imaging, such as magnetic resonance imaging (MRI) and lumbar punction with cerebrospinal fluid (CSF) examination. Moreover, both patients treated with intrathecal methotrexate had CNS relapse, which may suggest that it is not a sufficient option in this group.

In our study, we also aimed to establish prognostic factors for OS and PFS. Only CR was significantly associated with longer PFS (*p* = 0.001) and OS (*p* = 0.002). Additionally, nephrectomy was associated with longer OS (*p* = 0.0498), whereas hypoalbuminemia, creatinine > 1.5 mg/dL and Hgb < 10 g/dL were related to inferior OS. Previous reports are single-center observations, with small sample sizes, and were not powered enough to analyze prognostic factors relative to survival parameters. We did not observe a statistically significant impact of the histological sub-type on the survival parameters, likely due to the small size of our population and potentially differing therapeutical strategies for aggressive vs. indolent lymphomas.

According to previously published data, PRL typically has a poor clinical course, with the median OS of less than one year in all studies [7,19,20,24]. In our study, the median PFS was 2.11 years, while the median OS was 6.33 years. Despite treatment, the overall mortality rate was 17.1% during the entire follow-up period. Ten out of 32 patients died after treatment, and seven had relapsed by the end of treatment. The mortality rate was especially high in patients with bilateral disease, reaching 100%. Available case reports and case series have demonstrated a median OS of 6 months before the rituximab era [23,28]. Recent data show that OS and PFS may vary, as rituximab has significantly improved the outcome of patients with aggressive B-cell lymphoma, leading to better PFS and OS [7].

The main limitation of the present study is its retrospective nature and the limited number of patients included, which renders statistical analysis challenging. Additionally, changing therapeutical strategies over the period covered by the analysis (2000–2023), particularly the introduction of anti-CD20 monoclonal antibodies for the treatment of B-cell NHL, adds complexity to the interpretation of the results. For some patients, the available clinical records lacked a complete set of lab test data, which diminished the statistical power of the analysis. Finally, the presence of CD5 immunohistochemical staining and cell of origin in pathology results in DLBCL patients was another limitation. Nevertheless, we believe that our findings may be useful for clinical practice and may contribute to future studies aimed at developing recommendations for managing this very rare type of extranodal lymphoma, including the potential benefits of nephrectomy.

## 6. Conclusions

PRL is an extremely rare type of extranodal lymphoma, with a generally poor prognosis. Early detection and personalized treatment approaches in managing this rare and complex lymphoma subtype are of utmost importance. Current clinical practice is generally based on guidelines for nodal NHL, but our observations suggest that radical tumorectomy, when feasible, may improve the patients’ survival outcomes. There is hope that new therapeutical approaches, such as bi-specific monoclonal antibodies or chimeric antigen receptor T-cell (CAR T-cell) therapies, will further improve the outcomes for this challenging group of lymphomas. Further studies are needed to develop optimal treatment protocols and establish clearer guidelines for cases involving this rare organ involvement.

## Figures and Tables

**Table 1:** Characteristics of the study group. The data are presented as frequencies (percentage) unless otherwise specified.

Variable		N (%)
Number of patients		32 (100)
Median age at diagnosis (IQR)	63.3 (53.8–69.9)	
Gender	Female	19 (59.4)
	Male	13 (40.6)
Symptoms	Abdominal/Lumbar pain	9 (28.1)
	Systemic symptoms	7 (21.9)
	Incidentaloma	6 (18.8)
	Hematuria	5 (15.6)
	Fatigue	2 (6.3)
	Lymphadenopathy	1 (3.1)
	Hyponatremia	1 (3.1)
Diagnostic imaging used	CT	23 (71.9)
	USG	4 (12.5)
	PET/CT	2 (6.3)
	MRI	1 (3.1)
	Missing data	5 (15.6)
Site	Left kidney only	16 (50.0)
	Right kidney only	8 (25.0)
	Both kidneys	2 (6.3)
	Missing data	6 (18.7)
Lymph node involvement		19 (59.4)
Histologic diagnosis	DLBCL	18 (56.3)
	MZL	MZL: 7 (21.9) + MALT: 1 (3.1)
	FL	4 (12.5)
	HL	1 (3.1)
	HGBL	1 (3.1)
First-line treatment	Only immunochemotherapy * or chemotherapy **	21 (65.6)
	Radical nephrectomy with immunochemotherapy *	8 (25.0)
	Only radical nephrectomy	2 (6.3)
	Partial nephrectomy with immunochemotherapy *	1 (3.1)
Supportive treatment	G-CSF	15 (46.9)
	CNS prophylaxis	4 (12.5)
Response to the first-line treatment	CR	21 (65.6)
	PR	5 (15.6)
	PD	4 (12.5)
	Missing data	2 (6.3)
Laboratory results at diagnosis	Hgb < 10 g/dL	5 (15.6)
	Creatinine > 1.5 mg/dL	7 (21.9)
	Albumin < 35 g/L	10 (31.3)
	β-2M > 3.5 mg/L	18 (56.3)
	LDH > 225 U/L	7 (21.9)
Mean tumor size [cm]		9.5 ± 4.4
Maximum tumor size > 5 cm		18 (56.3)
CNS-IPI risk ***	Low	1 (5.6)
	Intermediate	12 (66.7)
	High	5 (27.8)
CNS relapse		5 (27.8)

* R-CHOP (cyclophosphamide, doxorubicin, prednisone, rituximab and vincristine) for DLBCL and HGBL patients, R-COP (cyclophosphamide, prednisone, rituximab and vincristine) for MZL and MALT patients. ** CHOP (cyclophosphamide, doxorubicin, prednisone and vincristine) for DLBCL patients, ABVD (doxorubicin, bleomycin, vinblastine and dacarbazine) for HL patients. *** Analyzed only for DLBCL patients (n = 18). β-2M—beta2-microglobulin, CNS—central nervous system, CR—complete response, CT—computer tomography, DLBCL—diffuse large B-cell lymphoma, FL—follicular lymphoma, G-CSF—granulocyte colony-stimulating factor, IQR—interquartile range, Hgb—hemoglobin, HGBL—high-grade B-cell lymphoma, HL—Hodgkin lymphoma, LDH—lactate dehydrogenase, MALT—mucosa-associated lymphoid tissue lymphoma, MRI—magnetic resonance imaging, MZL—marginal zone lymphoma, N—number of patients, PET—positron emission tomography, PD—progressive disease, PR—partial response, USG—ultrasonograph.

**Table 2:** Univariate and multivariate Cox regression analysis for progression-free survival and overall survival of patients with primary renal lymphoma. Factors with *p* < 0.1 were entered into multivariate analysis.

PFS								
Variable	Univariate				Multivariate			
	*p*-Value	HR	95% CI		*p*-Value	HR	95% CI	
			Lower	Upper			Lower	Upper
Age ≥ 65	0.0542	0.34	0.11	1.02	0.0701	0.30	0.08	1.10
Sex (M)	0.5981	1.32	0.48	3.64				
Surgery during treatment	0.5479	0.70	0.22	2.25				
DLBCL diagnosis	0.9794	0.99	0.35	2.74				
MZL diagnosis	0.5250	1.42	0.48	4.19				
WBC	0.7847	1.01	0.93	1.09				
ANC	0.9582	1.01	0.72	1.41				
PLT	0.8784	1.00	0.99	1.01				
Urea	0.2602	0.99	0.97	1.01				
LDH	0.6744	1.00	1.00	1.00				
β-2M	0.3288	1.19	0.84	1.67				
Creatinine > 1.5 mg/dL	0.0124	5.58	1.45	21.47	0.0042	10.20	2.08	50.09
Hgb < 10 g/dL	0.0602	3.08	0.95	9.92	0.0054	8.39	1.88	37.49
Hypoalbuminemia	0.2338	2.00	0.64	6.29				
**OS**								
**Variable**	**Univariate**				**Multivariate**			
	***p*-Value**	**HR**	**95% CI**		***p*-Value**	**HR**	**95% CI**	
			**Lower**	**Upper**			**Lower**	**Upper**
Age ≥ 65	0.2262	0.44	0.12	1.66				
Sex (M)	0.8288	0.88	0.27	2.90				
Surgery and treatment	0.0943	0.17	0.02	1.35	0.2140	0.27	0.03	2.15
DLBCL diagnosis	0.1503	2.66	0.70	10.06				
MZL diagnosis	0.1920	0.25	0.03	2.00				
WBC	0.6020	0.96	0.83	1.12				
ANC	0.1769	1.27	0.90	1.80				
PLT	0.1393	1.00	1.00	1.01				
Urea	0.7622	1.00	0.97	1.02				
LDH	0.4356	1.00	0.99	1.00				
β-2M	0.1615	1.53	0.84	2.78				
Creatinine > 1.5 mg/dL	0.1758	2.40	0.68	8.55				
Hgb < 10 g/dL	0.1061	3.04	0.79	11.69				
Hypoalbuminemia	0.0429	4.11	1.05	16.15	0.0356	5.44	1.12	26.38

ANC—absolute neutrocyte count, β-2M—beta2-microglobulin, DLBCL—diffuse large B-cell lymphoma, Hgb—hemoglobin, HR—hazard ratio, LDH—lactate dehydrogenase, M—male, MZL—marginal zone lymphoma, OS—overall survival, PLT—platelet count, PFS—progression-free survival, WBC—white blood count.

## Data Availability

The original contributions presented in this study are included in the article. Further inquiries can be directed to the corresponding author.
